# Magnetic properties of superparamagnetic nanoparticles loaded into silicon nanotubes

**DOI:** 10.1186/1556-276X-9-413

**Published:** 2014-08-21

**Authors:** Petra Granitzer, Klemens Rumpf, Roberto Gonzalez, Jeffery Coffer, Michael Reissner

**Affiliations:** 1 Institute of Physics, Karl Franzens University Graz, Universitaetsplatz 5, Graz 8010, Austria; 2 Department of Chemistry, Texas Christian University, Fort Worth, TX 76129, USA; 3 Institute of Solid State Physics, Vienna University of Technology, Wiedner Hauptstr. 8Vienna 1040, Austria

**Keywords:** Silicon nanotubes, Magnetic nanoparticles, Superparamagnetism, Iron oxide, Drug delivery

## Abstract

In this work, the magnetic properties of silicon nanotubes (SiNTs) filled with Fe_3_O_4_ nanoparticles (NPs) are investigated. SiNTs with different wall thicknesses of 10 and 70 nm and an inner diameter of approximately 50 nm are prepared and filled with superparamagnetic iron oxide nanoparticles of 4 and 10 nm in diameter. The infiltration process of the NPs into the tubes and dependence on the wall-thickness is described. Furthermore, data from magnetization measurements of the nanocomposite systems are analyzed in terms of iron oxide nanoparticle size dependence. Such biocompatible nanocomposites have potential merit in the field of magnetically guided drug delivery vehicles.

**PACS:**

61.46.Fg; 62.23.Pq; 75.75.-c; 75.20.-g

## Background

Porous materials with their substantial surface areas are versatile structures with specific properties of value for diverse fields such as photonics, catalysis, and therapeutics [1]. As an elemental semiconductor, porous silicon is a unique example of this type of material whose biocompatibility and biodegradability lend it great potential value to biomedical applications [2]. The complementary morphology of hollow silicon nanotubes (SiNTs) also provides opportunities in areas such as battery technology, photovoltaics, as well as drug delivery. SiNTs are tunable in their inner diameter as well as in their wall-thicknesses [3]. They provide a uniform structure compared to the dendritic pore growth of porous silicon in the target porous regime (30 to 90 nm pore diameter), and therefore, such structures are attractive for infiltration with nanoparticles or molecules (e.g., superparamagnetic (SPM) iron oxide nanoparticles of the form Fe_3_O_4_). In terms of possible candidates for loading, superparamagnetic Fe_3_O_4_ nanoparticles (NPs) also offer a low toxicity and thus can be applied to diverse uses in biomedicine, e.g., for hyperthermia, NMR imaging, and functionalization with anti-cancer agents [4].

In this work, SiNTs are infiltrated with Fe_3_O_4_ NPs to achieve a nanocomposite system which can, in the long term, be considered for use as a magnetic-assisted drug delivery vehicle. Previously, porous silicon loaded with iron oxide NPs of different sizes has been investigated with the cytocompatibility of this system showing encouraging results [5]. The cytocompatibility of SiNTs has also been recently evaluated [6]. In the following work, the infiltration of Fe_3_O_4_ NPs into SiNTs of different wall thicknesses is described and the fundamental magnetic properties of these composites investigated as a function of the Fe_3_O_4_-nanoparticle size.

## Methods

Silicon nanotubes were fabricated by a multistep process previously described [3] involving deposition of silane (SiH_4_) on preformed ZnO nanowire array templates on F-doped tin oxide (FTO) glass or Si wafer segments, followed by sacrificial etching of the ZnO phase resulting in the desired nanotube product. Hollow nanotube inner diameter is adjustable by size selection of the initial ZnO nanowire template, while shell thickness control is achieved by concentration/duration of silicon deposition. In these experiments, SiNTs with 10-nm wall thickness are obtained at 530°C with a 5-min Si deposition time, and SiNTs with 70-nm wall thickness are obtained at 580°C with a 5-min Si deposition time. Internal nanotube diameter is dependent on ZnO nanowire diameter, which in the experiments described here, is fixed at 50 nm. The wall thickness determines the dissolution of the material in vitro and thus is of importance for controlled drug release (vide infra).

Iron oxide NPs have been prepared by a known route utilizing decomposition of an iron complex at high temperature [7]. NPs of different sizes (4 and 10 nm) are infiltrated into SiNTs with 10- and 70-nm wall thicknesses. The infiltration process performed at room temperature is supported by a magnetic field to assure optimal filling of the nanotubes. The infiltration process has been optimized with respect to the wall-thickness of the SiNTs and the size of the NPs used.

For the case of relatively thick-walled nanotubes (70 nm), the loading of Fe_3_O_4_ NPs is readily achieved by initial removal of the SiNT film from the underlying substrate (such as FTO glass) and placing it face down on top of a Nd magnet with a piece of filter paper in between. Fe_3_O_4_ NPs (oleic acid terminated, hexane solution) at a concentration of 7 mg/mL are added dropwise, followed by rinsing the infiltrated sample with acetone several times, and allowed to air dry.

For the thin-walled SiNT variant (approximately 10 nm), the infiltration process of Fe_3_O_4_ NPs in thin shell thickness SiNTs is accomplished by placing the SiNTs attached to the substrate (e.g., silicon wafer) also on top of a Nd magnet. The Fe_3_O_4_ NPs are added dropwise (also at a concentration of 7 mg/mL), and the infiltration process is accomplished by diffusion of the nanoparticles through the side porous wall of the SiNT. For the case of Fe_3_O_4_ nanoparticles that are 10 nm in diameter, the SiNT sidewall pore dimensions are insufficient to permit loading by diffusion through this orifice and thus the SiNT film must be removed from the substrate prior to loading of this sample.

Magnetic measurements were performed with a vibrating sample magnetometer (VSM; Quantum Design, Inc., San Diego, CA, USA). Magnetization curves of the samples have been measured up to a field of 1 T, and the temperature-dependent investigations have been carried out between *T* = 4 and 300 K. Scanning electron micrographs (SEM) were measured using a JEOL FE JSM-7100 F (JEOL Ltd., Akishima-shi, Japan), with transmission electron micrographs (TEM) obtained with a JEOL JEM-2100.

## Results and discussion

**Figure 1:**
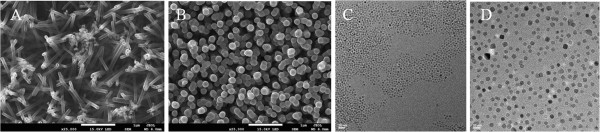
**FE-SEM images of SiNT array and TEM images of Fe**_**3**_**O**_**4 **_**NPs.** FE-SEM images of **(A)** SiNT array with 10-nm wall thickness and **(B)** SiNT array with 70-nm wall thickness. TEM images of **(C)** 4-nm Fe_3_O_4_ NPs and **(D)** 10-nm Fe_3_O_4_ NPs.

Silicon nanotubes (SiNTs) are most readily fabricated by a sacrificial template route involving silicon deposition on preformed zinc oxide (ZnO) nanowires and subsequent removal of the ZnO core with a NH_4_Cl etchant [3]. In the experiments described here, we focus on the infiltration of Fe_3_O_4_ nanoparticles into SiNTs with two rather different shell thicknesses, a thin porous variant with a 10-nm shell (Figure 1A) or a very thick 70-nm sidewall (Figure 1B). In terms of Fe_3_O_4_ nanoparticles, two different sizes were used for infiltration: relatively monodisperse nanocrystals with a mean diameter of 4 nm (Figure 1C), and a larger set of Fe_3_O_4_ nanocrystals of 10-nm average diameter and a clearly visible broader size distribution (Figure 1D).

**Figure 2:**
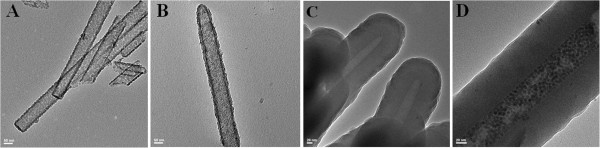
**TEM images of SiNTs. (A)** SiNTs with 10-nm wall thickness - empty; **(B)** SiNTs with 10-nm wall thickness filled with 4-nm Fe_3_O_4_ NPs; **(C)** SiNTs with 70-nm wall thickness - empty; and **(D)** SiNTs with 70-nm wall thickness filled with 4-nm Fe_3_O_4_ NPs.

The incorporation of superparamagnetic nanoparticles of Fe_3_O_4_ into hollow nanotubes of crystalline silicon (SiNTs) can be readily achieved by exposure of relatively dilute hydrocarbon solutions of these nanoparticles to a suspension/film of the corresponding nanotube, the precise details of which are dependent upon the shell thickness of the desired SiNT. For SiNTs possessing porous sidewalls (present when the nanotube thickness is approximately 12 nm or less), the voids present can be exploited to permit infiltration of the Fe_3_O_4_ nanoparticles through simple diffusion (if the nanoparticles are relatively small). For Si nanotubes with solid continuous sidewalls (as with the 70-nm-thick SiNTs studied here), the nanotubes must be physically removed from their underlying growth substrate, effectively ‘uncapping’ the SiNT array and allowing facile infiltration of Fe_3_O_4_ nanoparticles under the assistance of a simple Nd magnet. In either case, dense conformal loading of the Fe_3_O_4_ into a given nanotube interior can be accomplished (Figure 2).

The purpose of fabricating such a magnetic nanocomposite is its applicability in biomedicine as a magnetic-guided drug delivery vehicle. A key requirement of such a system is a low blocking temperature (*T*_B_) which is defined by the transition between superparamagnetic (SPM) behavior and the blocked state of the nanocomposite. *T*_B_ has to be far below room temperature, which entails a missing magnetic remanence. So above *T*_B_, the system offers no magnetic remanence if the external field is switched off. From temperature-dependent magnetization measurements, the transition temperature between SPM behavior and blocked state has been extracted. The so-called blocking temperature *T*_B_ depends strongly on the particle size of the infiltrated iron oxide NPs and on the distance between the particles within the tubes. To obtain *T*_B_ of the nanotubes with different infiltrated NPs, zero field cooled/field cooled (ZFC/FC) magnetization measurements have been performed. For this purpose, the sample is first cooled down from room temperature to *T* = 4 K without an external magnetic field. Then, a low magnetic field of *H* = 500 Oe is applied and the magnetization measured up to *T* = 300 K and subsequently down to *T* = 4 K.

**Figure 3:**
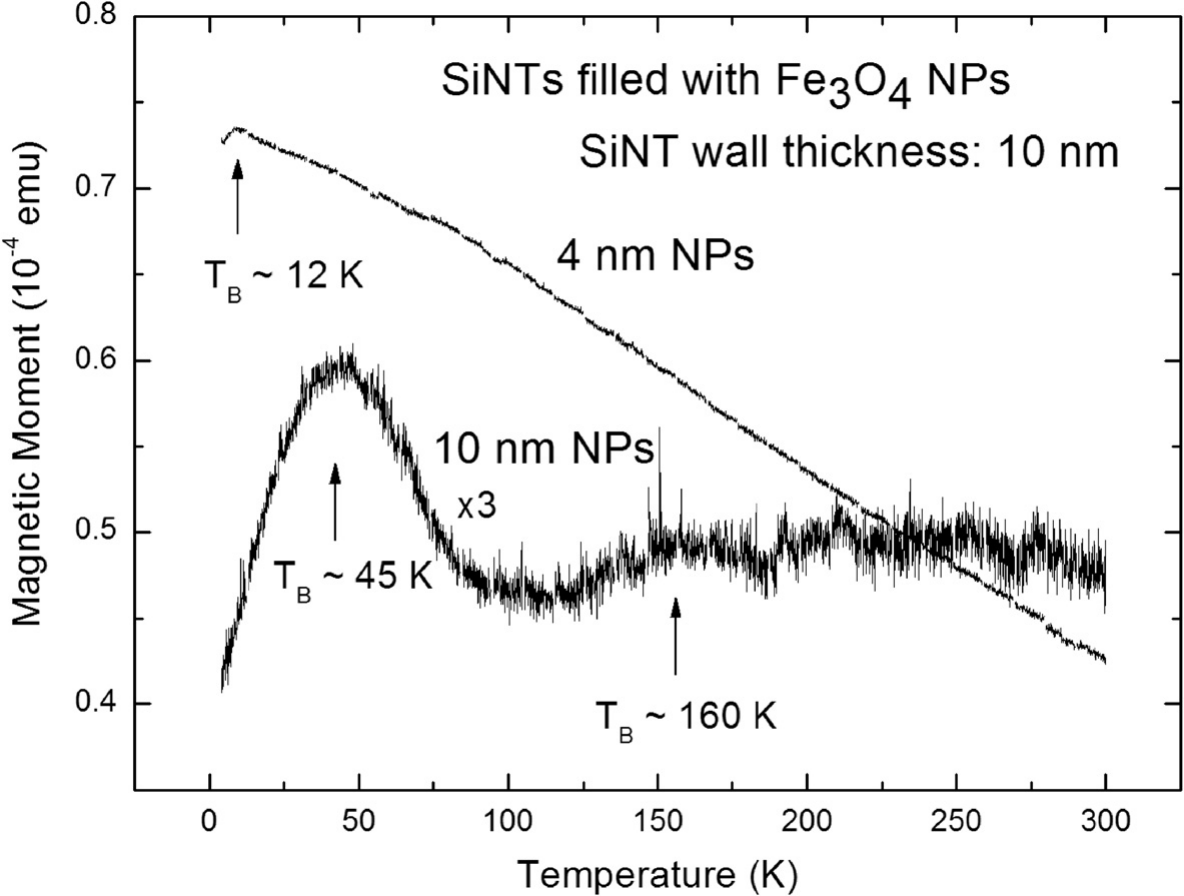
**ZFC/FC measurements of SiNTs (wall thickness 10 nm) filled with iron oxide NPs of 4 and 10 nm in size.** One can see that the sample containing 4-nm NPs offers a *T*_B_ of 10 K, whereas the sample with 10-nm NPs shows two peaks at 45 and 160 K.

**Figure 4:**
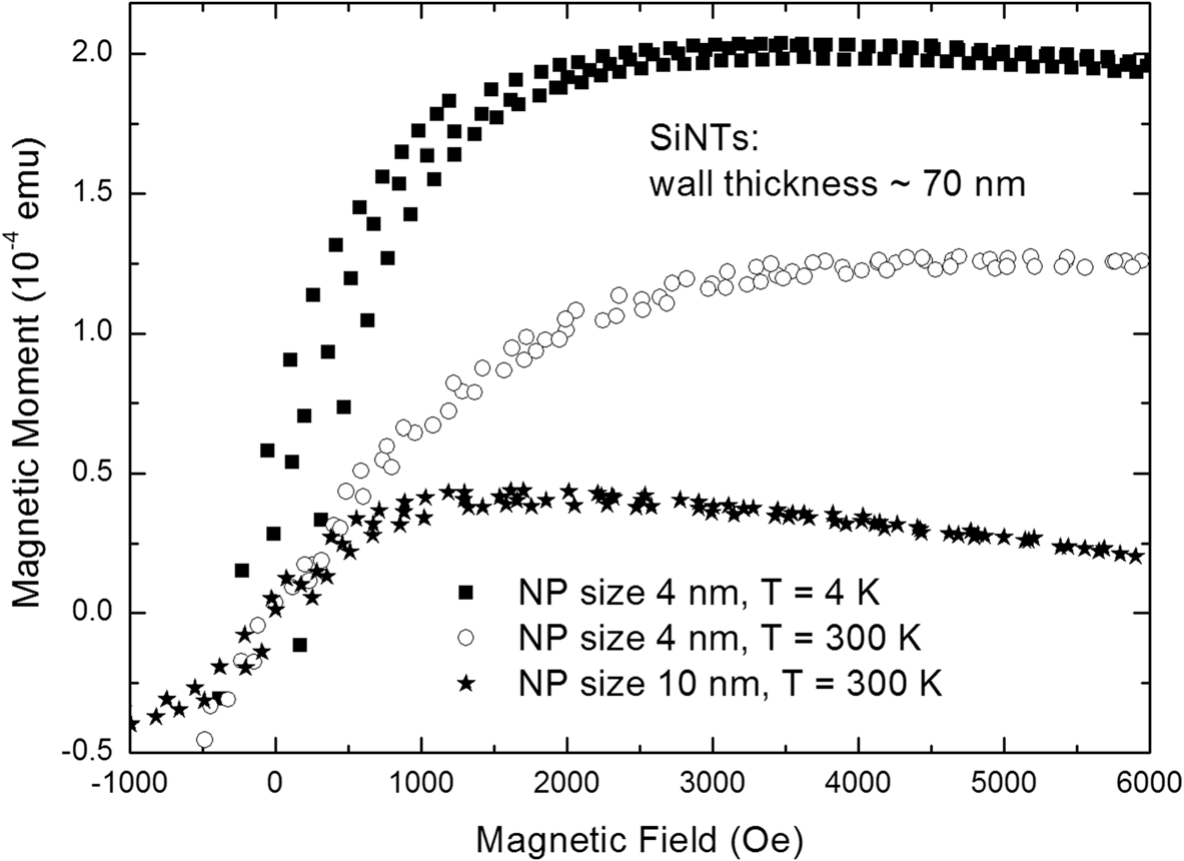
**SiNT hysteresis curves.** Hysteresis curves of SiNTs offering a wall thickness of about 70 nm filled with iron oxide NPs of 4 nm (squares, measured at *T* = 4 K; circles, measured at *T* = 300 K) and 10 nm (stars, measured at *T* = 300 K).

In these initial studies, we report the different blocking temperatures for Fe_3_O_4_ nanoparticles of either 4 or 10 nm infiltrated into SiNTs containing 10- or 70-nm thick walls (Table 1). Remarkably low *T*_B_ values of 12 K are found for the 4-nm Fe_3_O_4_ nanoparticles loaded into both the 10-nm as well as 70-nm thick SiNTs, indicating that the iron oxide particles do not interact magnetically. For the larger 10-nm-diameter Fe_3_O_4_ nanoparticles loaded into either the 10- or 70-nm thick SiNTs, two to three different discrete blocking temperatures are observed for a given nanotube sample (all well below room temperature) (Figure 3), consistent with a broader distribution of nanoparticle sizes in the iron oxide (as observed in the TEM image of these nanoparticles in Figure 1D). Further evidence of a missing remanence above the *T*_B_ can be seen by analyzing measurements of field-dependent magnetization. Figure 4 shows hysteresis curves of SiNTs with 70-nm wall thickness loaded with 4- and 10-nm Fe_3_O_4_ NPs measured below and above *T*_B_. The measurements at low temperatures (*T* = 4 K) show a coercivity *H*_C_ of about 200 Oe, whereas at temperatures above *T*_B_ (*T* = 300 K), the coercivity is nearly vanished (*H*_C_ ~ 50 Oe).

**Table 1:** Summary of the various blocking temperatures, magnetic remanence, and coercivities gained by filling of SiNTs with iron oxide NPs of different sizes

	**NP size**	
	**4 nm**	**10 nm**
*T*_B_ (K)		
10-nm shell SiNTs	12	45/160
70-nm shell SiNTs	12	30/125/160
70-nm shell SiNTs, remanence *M*_R_ (emu)		
*T* = 4 K	0.75 × 10^-4^	0.55 × 10^-4^
*T* = 300 K	0.01 × 10^-4^	0.01 × 10^-4^
70-nm shell SiNTs, coercivity *H*_C_ (Oe)		
*T* = 4 K	200	220
*T* = 300 K	50	60

These initial investigations suggest that the loading of SiNTs with different wall thicknesses retain a heavily suppressed blocking temperature (*T*_B_) far below room temperature, a promising result. A systematic investigation of the nanotube wall thickness on blocking temperature is currently under evaluation, but studies to date suggest that the magnetic properties can be tuned by the filling of the SiNTs independent of the nanotube wall thickness. Given our previous observation of thickness-dependent dissolution behavior for these nanotubes in aqueous media [3], this parameter can be paired with a target blocking temperature and selected based on the desired degradation window in vivo.

## Conclusions

Silicon nanotubes filled with superparamagnetic iron oxide NPs were investigated with respect to a possible utilization as magnetically guided drug delivery vehicle. The magnetic properties were found to be dependent upon the NP size but relatively insensitive to the morphology of the nanostructured Si host. The blocking temperature is very low for all investigated samples which enables a closely packed filling of the nanotubes to achieve a magnetic moment as high as possible. These results are encouraging and fulfill the preconditions for applicability of these semiconducting nanotubes in biomedicine.

## Competing interests

The authors declare that they have no competing interests.

## Authors’ contributions

RG fabricated the SiNT samples, their loading with Fe_3_O_4_ nanoparticles, and microstructural characterization. PG and KR performed the magnetic measurements. PG, KR, RG, JC, and MR discussed the data and prepared the manuscript. All authors read and approved the final manuscript.

## References

[B1] Nanoporous materials In Science and Engineering 2004 Singapore: World Scientific Press: Edited by Lu GQ, Zhao XS

[B2] Canham LT Adv Mater 1995 9 1033 1037 10.1002/adma.19950071215

[B3] Huang X Gonzalez-Rodriguez R Rich R Gryczynski Z Coffer JL Chem Commun 2013 9 5760 10.1039/c3cc41913d 23695426

[B4] Gupta AK Gupta M Biomaterials 2005 9 3995 4021 10.1016/j.biomaterials.2004.10.012 15626447

[B5] Granitzer P Rumpf K Tian Y Akkaraju G Coffer J Poelt P Reissner M Appl Phys Lett 2013 9 193110 10.1063/1.4807421

[B6] Tian Y Gonzalez R Akkaraju G Coffer J Presentation at Porous Semiconductors Science and Technology 2014 Spain: Alicante-Benedorm Abstract 06-O-15

[B7] Roca AG Costo R Rebolledo AF Veintemillas-Erdaguer S Tartaj P Gonzalez Carreno T Morales MP Serna CJ J Phys D: Appl Phys 2009 9 224002 10.1088/0022-3727/42/22/224002

